# The accuracy of recording malaria rapid diagnostic test (RDT) results in public health facilities in Benin; results from the MaCRA project

**DOI:** 10.1186/s12936-026-05871-7

**Published:** 2026-03-28

**Authors:** Idelphonse Ahogni, Hospice Avanon, Corneille Hueha, Augustin Kpemasse, Julien Aissan, Cyriaque Affoukou, Manfred Accrombessi, John J. Aponte, Emily Hilton, Shawna Cooper, Kevin Griffith, Michael Humes, Kim A. Lindblade, Corine Ngufor

**Affiliations:** 1 Centre de Recherche Entomologique de Cotonou, CREC, Cotonou, Benin; 2 Programme National de Lutte contre le Paludisme (PNLP), Cotonou, Benin; 3 PMI Insights Project/PATH, Seattle, WA USA; 4 Audere, Seattle, WA USA; 5 United States President’s Malaria Initiative, Washington, DC USA; 6 London School of Hygiene and Tropical Medicine, LSHTM, London, UK; 7 Liverpool School of Tropical Medicine, LSTM, Liverpool, UK

**Keywords:** Malaria, Rapid diagnostic test, RDT, Healthcare workers, Diagnostic accuracy, Benin, Surveillance, Case management, KAPB survey

## Abstract

**Background:**

Accurate interpretation and recording of malaria rapid diagnostic tests (RDTs) are critical for case management and surveillance in malaria-endemic settings. In Benin, where over 90% of malaria diagnoses rely on RDTs, concerns remain about the accuracy of the reporting and recording of RDT results. This study assessed the fidelity of RDT recording by healthcare workers (HCWs) in public health facilities and explored associated factors.

**Methods:**

A six-month mixed-methods, prospective observational study was conducted in 16 public health facilities across two departments in Benin. For each RDT performed, an image was captured using a digital RDT reader (HealthPulse, Audere, Seattle, WA USA) and independently interpreted by an external trained panel. HCW-recorded results were compared to panel interpretations. A knowledge, attitudes, practices, and beliefs (KAPB) survey and structured observations of RDT performance were conducted, alongside in-depth interviews with selected HCWs.

**Results:**

Of 35,720 RDTs assessed, overall agreement between HCW and reference panel interpretations was 94.3% (Cohen’s kappa = 0.88). Results misrecorded as positive (5.0%) were more frequent than results misrecorded as negative (0.7%). Agreement varied by patient age, HCW experience, and facility characteristics. Accuracy was highest with children under 5 years (96.7%) and lowest with patients over 15 years (91.6%). HCWs with ≥ 10 years of experience, and access to electricity and internet performed better. From 226 HCWs surveyed, 89.4% believed a patient with malaria could have a negative RDT, though only 19.5% supported treating such cases with antimalarials. While most HCWs were proficient in performing RDTs, only 40.5% waited the recommended time before reading results, and glove use was low (15.6%) highlighting safety gaps. RDT use was primarily motivated by adherence to guidelines (60.2%), rather than patient or supervisor expectations. Qualitative interviews highlighted contextual challenges including workload, lighting conditions in health facilities, and resource constraints.

**Conclusion:**

HCWs in Benin showed high accuracy in interpreting and reporting malaria RDT results, likely supported by recent nationwide RDT cassette validations. Performance was strongest among those with more experience, training, and adequate infrastructure. However, negative results misrecorded as positive, especially in adult patients, remains a concern. Targeted training and supportive supervision may help strengthen confidence in negative results and improve overall diagnostic accuracy.

**Supplementary Information:**

The online version contains supplementary material available at 10.1186/s12936-026-05871-7.

## Background

Malaria remains a leading cause of mortality among children under five and a major contributor to morbidity in adults in Benin. In 2022 alone, an estimated 5 million malaria cases and over 11,000 related deaths were reported [1]. To address this burden, Benin’s national malaria policy promotes early diagnosis and prompt treatment at all levels of the healthcare system in line with World Health Organization (WHO) guidance [2]. This includes the use of rapid diagnostic tests (RDTs) or microscopy for confirming all suspected cases of malaria prior to treatment. Patients who test positive are expected to receive a full course of WHO-recommended antimalarial treatment. Conversely, those who test negative should not be prescribed antimalarials but should undergo thorough clinical evaluation to identify alternative causes of fever.

Malaria diagnosis in Benin relies heavily on RDTs, which account for over 90% of all confirmed malaria cases [3, 4]. RDTs are simple, easy-to-use immunochromatographic devices that detect parasite-specific antigens or enzymes in the blood, targeting either the *Plasmodium* genus or specific species [5]. They are accessible across all levels of the health system in Benin and are especially critical in peripheral health facilities where access to microscopy may be limited. Since their introduction in 2008, RDTs have been provided free of charge within the public health sector, contributing significantly to improved diagnostic coverage and case management across the country [4]. Patients presenting at public health facilities with a body temperature of 37.5 °C or higher, or with a reported history of fever within the previous 48 h, are routinely tested for malaria using RDTs. Following administration of the RDT, patient information (including clinical presentation, diagnostic test results, and treatment provided) is recorded in the health facility registers and later entered into the national Health Management Information System (HMIS).

Given the reliance on RDTs for diagnosis, effective malaria surveillance in Benin therefore hinges on the proper management of RDT results. Although RDTs generally demonstrate high diagnostic performance, the quality of malaria surveillance data in Benin remains susceptible to challenges associated with their routine implementation [6]. Key issues include healthcare workers’ (HCWs) ability to correctly administer and interpret test results, adherence to national treatment guidelines based on RDT outcomes, and the accurate recording of diagnostic and treatment information in patient registers and national HMIS reporting systems. These data form the backbone of the HMIS informing public health decision-making, guiding malaria control strategies, and supporting the efficient allocation of resources.

Ensuring accurate recording of RDT results is essential for tracking malaria trends and supporting timely, evidence-based policy responses at both national and sub-national levels. However, surveys conducted by the National Malaria Control Programme (NMCP) of Benin in selected public health facilities in 2019 revealed significant issues with HCWs’ adherence to and recording of negative malaria RDT results [7]. In many instances, HCWs recorded patients with negative test outcomes as malaria-positive and treated them with artemisinin-based combination therapies (ACTs), opting to rely on presumptive diagnosis rather than test outcomes. In response, the Ministry of Health in 2023 launched a decentralized programme to validate malaria data on a monthly basis [8]. This initiative involves cross-checking data using primary sources from health facilities, including patient registers and used RDT cassettes, before the information is entered into the HMIS. Such issues are not unique to Benin and have been documented in several countries across sub-Saharan Africa [6, 9–11]. They compromise the accuracy of malaria surveillance data and can lead to inappropriate clinical management, including the unnecessary use of antimalarial drugs in patients who do not have malaria and increased drug resistance risk.

To better understand and address these challenges, we conducted a mixed-methods, prospective observational study in public health facilities in Benin as part of the broader multi-country Malaria RDT Capture and Reporting Assessment (MaCRA project)[12]. The study aimed to evaluate the accuracy of RDT result reporting by comparing outcomes recorded in health facility registers with those independently verified by an external panel of trained reviewers. Over a six-month period, RDT results captured in routine facility registers were compared to results interpreted by the external panel reviewing images of the RDTs, which had been taken using a smartphone application (HealthPulse, Audere, Seattle, WA USA). The study also sought to identify key factors contributing to reporting discrepancies within the Beninese context. Here, we present a descriptive analysis highlighting key trends, patterns, and variations observed in HCWs’ accuracy of recording malaria RDT results across health facility, patient and HCW characteristics in Benin.

## Methods

### Study sites in Benin

**Fig. 1:**
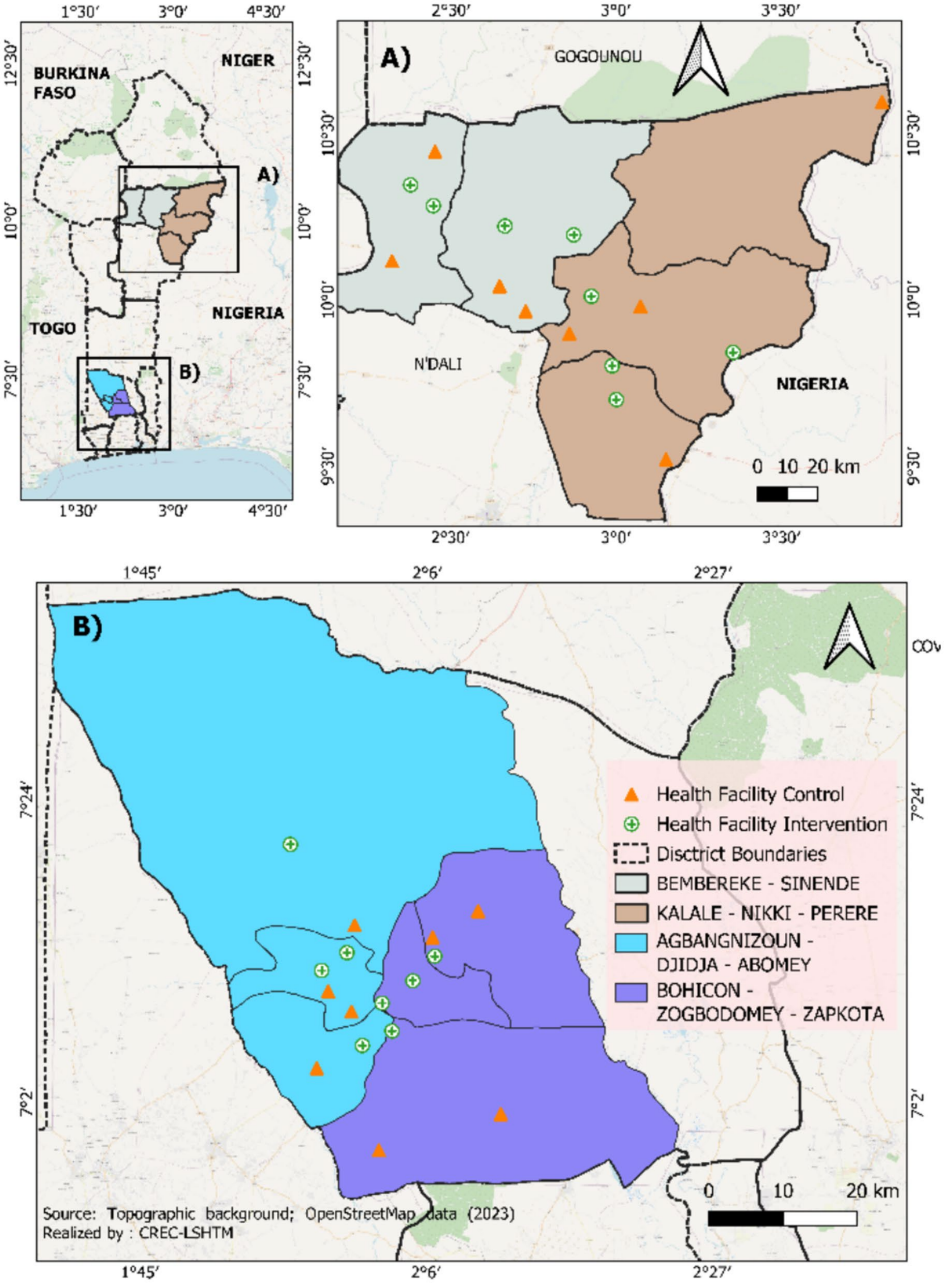
Map of study sites and health facilities involved in study

The study was conducted between June and December 2023 in four Health Zones across two Departments in Benin; one located in the north, where malaria transmission is seasonal (Borgou), and one in the south, where transmission is perennial (Zou). In collaboration with national stakeholders and the National Malaria Control Programme (NMCP), the Health Zones of Bembèrèkè-Sinendé and Nikki-Kalalé-Pèrèrè in the Borgou Department were selected to represent the northern site. For the southern site, the Health Zones of Bohicon-Za-Kpota-Zogbodomey and Djidja-Abomey-Agbangnizoun in the Zou Department were chosen (Fig. 1).

Selection of the study sites was based on several criteria; the absence of major ongoing malaria control interventions (such as seasonal malaria chemoprevention [SMC] and perennial malaria chemoprevention [PMC]) that could confound study outcomes; the presence of a sufficient number of public health facilities; patient volume; a consistently high RDT test positivity rate (TPR) over recent years; availability of routine malaria data for at least nine months per year over the past three years; and logistical and financial feasibility, including site accessibility. The selected Health Zones exhibited persistently high RDT TPRs with minimal variation between 2019 and 2021 based on HMIS data, further supporting their suitability for inclusion in the study.

A total of 32 public health facilities (16 in the northern site and 16 in the southern site) were selected (Fig. 1). Eligible health facilities were those with at least two to three years of malaria reporting to the national HMIS, with data available for at least nine out of twelve months per year and a minimum of 50 RDTs per month. Facilities within each administrative district were stratified into four categories based on median values of patient volume (high/low) and average TPRs (high/low). From each of the four strata in each district, one health facility was randomly selected to participate in the study, resulting in 16 study facilities per site. Additionally, one control facility was selected from each stratum in each district; these control sites did not participate in study activities and were used to assess the potential impact of the study on TPR trends in a separate interrupted time series analysis. To ensure uninterrupted diagnostic and treatment services, all study and control facilities were supplied with adequate stocks of RDTs and antimalarial medicines throughout the study period as part of the routine health services.

### Overview of study design

A summary of the study methodology, which is described in detail elsewhere [12], is provided below:

*Health facility survey:* At the beginning of the study, each health facility was surveyed through interviews and direct observation to assess operational capacity. The survey documented geolocation, staff numbers (especially those performing RDTs), availability of registers, guidelines, equipment, diagnostics, antimalarial medicines, and any recent stockouts. Infrastructure such as electricity and internet connectivity was also reviewed.

*Knowledge, attitudes, perceptions and behavior (KAPB) and RDT proficiency:* At baseline, a survey was administered to all HCWs in the evaluation health facilities who were currently involved (or likely to be involved) in malaria rapid diagnostic testing, interpretation of results, treatment decisions, or documentation of RDT outcomes. Informed consent was obtained from all participants. The survey gathered information on HCWs’ training and professional experience, knowledge of malaria transmission and case management, attitudes toward RDTs, perceptions of RDT accuracy, prescribing behaviors, and perceived norms related to malaria diagnosis, treatment, and surveillance practices. HCWs were observed while performing malaria RDTs and assessed using a standardized checklist. The evaluation focused on key areas including procedural steps, accuracy in test administration and interpretation, and adherence to biosafety protocols.

*Healthcare worker interpretations of RDT results:* Over the six-month study period, the accuracy of HCWs’ recording of malaria RDTs was evaluated by comparing results recorded in facility registers with those assessed by an independent panel of trained reviewers. Panelists were trained on both good and poor-quality photos and were required to pass a test that involved correctly interpreting and labeling a set of 50 images [12]. The panel reviewed images of completed RDTs captured using the HealthPulse smartphone digital RDT reader application, developed by Audere (Seattle, WA USA). Data collectors used project-issued smartphones to capture RDT images, including the HCW’s unique ID, their recorded interpretation, and basic patient details such as age and sex. To accurately link images with corresponding register entries, barcodes were affixed to both the RDT cassette and the matching register line. The external panel was blinded to all patient and facility information. They classified each RDT image as positive or negative based solely on the presence or absence of control and test lines, following the manufacturer’s guidelines. Patient care was managed entirely by health facility staff based on their own RDT interpretations, in line with national case management protocols. Data collectors had no interaction with patients and did not influence clinical decision-making.

*In-depth interviews:* At the end of the study, in-depth interviews were conducted with a random selection of 16 HCWs demonstrating both high (8) and low (8) agreement with the reference panel. These aimed to explore key drivers and root causes of discrepancies in RDT performance, documentation, and adherence to diagnostic results. HCWs were asked open-ended questions exploring their experiences with malaria RDTs, including their understanding of procedures, challenges in interpreting results, and responses to cases where clinical symptoms contradict RDT outcomes. They also discussed how they manage inconclusive results, stockouts of RDTs and supplies, and high patient volumes. Finally, HCWs were asked about the influence of the monthly malaria RDT validation exercise in Benin on their diagnostic and reporting practices.

### Sample size

The primary objective of the study was the degree of agreement between the RDT results reported in the health facility register by HCWs and the panel RDT results measured using Cohen’s kappa which corrects for chance agreement. The sample size was thus based on the precision for estimating Cohen’s kappa for an individual HCW [12]. A range of kappa scores between 0.7 and 0.9 was assumed and the number of RDTs needed for different levels of precision calculated. The probability of a positive rating was estimated by the TPR. Assuming TPRs between 30 and 70%, the maximum sample size needed to calculate 95% CI with a width of no more than 0.2 was 236 RDTs. It was estimated that individual HCWs would likely interpret between 40 and 80 RDTs each month, for a possible range of 320–480 observations per HCW over the course of the evaluation, which would be sufficient for a precise measurement of Cohen’s kappa at both the HCW and health facility level. The number of study facilities included was fixed at 16 due to budget limitations.

### Data management and analysis

All questionnaires were digitized and deployed on smartphones using the Open Data Kit (ODK) platform. Electronic data were securely stored in password-protected database systems, with access limited to designated project staff. Data were exported from KoboToolbox and the HealthPulse application into centralized databases. Standardized scripts were developed in R (R Foundation for Statistical Computing, Vienna, Austria) to generate cleaned and analysable datasets. Statistical analyses were performed using R. The accuracy of HCWs’ interpretation of malaria RDTs was assessed by measuring agreement between the RDT results they recorded in health facility registers and those determined by the external panel. Agreement was quantified using Cohen’s kappa statistic, which accounts for agreement occurring by chance. Discrepancies, where HCWs recorded a result different from that reported by the panel, were classified as results that were either misrecorded as positive or negative (Table 1).

**Table 1:** Illustration of cross-tabulation of HCW and external panel RDT results

HCW RDT result	Panel RDT result		
	Positive	Negative	Invalid
Positive	A	(Misrecorded as positive) B	(Misrecorded as positive) E
Negative	(Misrecorded as negative) C	D	(Misrecorded as negative) F

The agreement between HCW and panel RDT results was calculated by health facility and a random effects meta-analysis used to summarize results according to various factors at the health facility and patient level. A bespoke R function was developed to summarize Cohen’s kappa scores by calculating a great mean and weighting by the inverse of the standard error (SE) for each health facility [16].

### Ethical considerations

The study received ethical approval from the Comité National d’Éthique pour la Recherche en Santé (CNERS) under the Ministry of Health in Benin (Approval No. CNERS019/2023), as well as from the Western Institutional Review Board (WCG IRB). Written informed consent was obtained from HWCs participating in the study. Patient consent was not required, as RDT images were anonymized and data extracted from health facility registers constituted secondary, non-identifiable information.

## Results

### Study profile

**Fig. 2:**
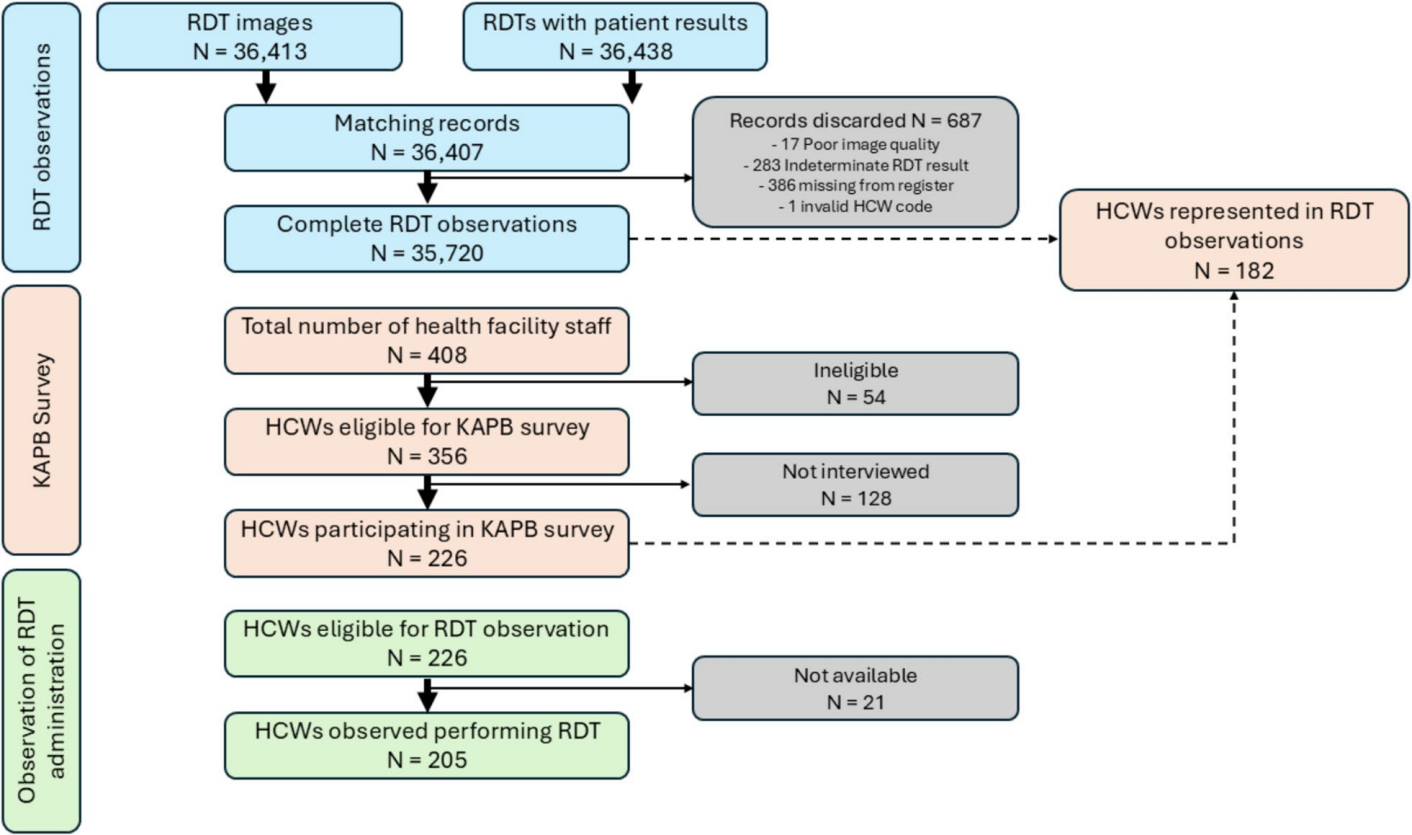
Study profile

During the six-month study period, a total of 36,413 RDT images and data for 36,438 patients who underwent malaria RDT testing were captured using the HealthPulse app. Among these, 36,407 matching records were successfully identified (Fig. 2). There were 687 records excluded due to various issues: 17 did not meet image quality standards, 283 had indeterminate results as interpreted by the external panel, 386 were missing from the health facility register, and 1 had an invalid HCW identification code, resulting in 35,720 observations for analysis.

Of the 408 HCWs present across the study facilities, 354 (86.8%) were eligible to participate in the KAPB survey. The remaining 54 HCWs were not involved in RDT administration. Among the eligible HCWs, 226 (63.8%) completed the KAPB survey, and 205 (57.9%) were observed performing an RDT using the standardized checklist. A total of 128 (36.2%) HCWs did not participate, primarily due to absence during the study period. By the end of the study, complete RDT observation data were available for 35,720 RDTs performed and interpreted by 182 (51.4%) HCWs.

### Characteristics of study health facilities

Among the 16 health facilities included in the study, malaria prevalence data derived from malariaAtlas, (an R package developed by the Malaria Atlas Project to facilitate accessing their data[13]) ranged from 30 to 39% in 15 facilities, while one facility had a lower prevalence of 20–29%. Four facilities were equipped with laboratories; however, all 16 facilities recorded malaria RDT results exclusively in outpatient and antenatal clinic registers. Job aids for performing RDTs were available in 11 facilities, and malaria case management guidelines were present in 14 (Additional file 1: Table S1). Most facilities (11 out of 16) had more than five HCWs who routinely performed malaria RDTs. There were no recent shortages of first- or second-line antimalarial medications in any of the facilities, although minor stockouts of antibiotics were observed. Only one facility reported a temporary shortage of RDTs at baseline. Throughout the study period, a total of only seven days of RDT stockouts were recorded across all sites.

With regard to medical equipment, all facilities had thermometers and weighing scales, but only three had timers specifically for RDTs. Microscopes for malaria diagnosis were available exclusively in the four facilities with laboratory capacity. As for infrastructure, 13 facilities had internet access, 8 had piped water, and 2 lacked an electricity supply (Additional file 1: Table S1).

### Characteristics of healthcare workers

The median number of HCWs who participated in the KAPB survey per health facility was 11.5 (IQR: 7.75–17.5). Among the 226 participating HCWs, 71.2% were women and approximately 70% were under the age of 40 (Table 2). Fewer than 8% were over 50 years old. The largest occupational group was medical auxiliary staff (42.9%), followed by nurses (19.9%) and students/interns/volunteers (18.1%). Only 8 medical doctors (3.5%) and 2 community health workers (0.9%) were included. Educational attainment was generally low: 64.2% had completed only primary education or less, while just 14.2% had reached university level. Nearly 40% had more than 10 years of experience, whereas 17.3% had one year or less. All HCWs who participated in the survey had experience performing RDTs and were also involved in related tasks. More than 80% were responsible for determining diagnoses and recording results in patient cards and registers.

**Table 2:** Characteristics of healthcare workers

Characteristic	Value	Frequency	Percentage
Sex	Female	161	71.2
	Male	65	28.8
Age	< 30 years	89	39.4
	30–39 years	69	30.5
	40–49 years	50	22.1
	50–59 years	17	7.5
	60 + years	1	0.4
Occupational category	Community health worker	2	0.9
	Medical auxiliary staff	97	42.9
	Medical doctor	8	3.5
	Midwife	31	13.7
	Non-medical staff	2	0.9
	Nurse	45	19.9
	Student, intern or volunteer	41	18.1
Highest qualification achieved	Primary school or below	145	64.2
	Secondary school	49	21.7
	University	32	14.2
Years of experience	0–1 years	39	17.3
	2–4 years	57	25.2
	5–9 years	42	18.6
	10 + years	88	38.9
Hours worked in a week	> = 50 h	181	80.1
	0–19 h	4	1.8
	20–39 h	5	2.2
	40–49 h	36	15.9
Tasks performed related to RDTs	Performs RDTs	226	100.0
	Performs microscopy	23	10.2
	Determines diagnosis/treatment	204	90.3
	Dispenses medicine	100	44.2
	Writes results in patient cards	192	85.0
	Writes information in registers	195	86.3
	Completes monthly record forms	88	38.9
Frequency of performing RDTs	Very often (every day)	195	86.3
	Once in a while to often	22	9.7
	Never	9	4.0
Frequency of recording RDT results	Very often (every day)	180	79.6
	Once in a while to often	16	7.1
	Never	30	13.3

Regarding training in RDT procedures, only half of the HCWs reported having received offsite training, and just 18.6% of these had received such training within the past year. Similarly, 45% reported receiving onsite training, with 38% indicating that this occurred in the past year. A greater proportion (68%) stated that they had been observed by a supervisor while performing an RDT at some point, although only 35.8% reported that such supervision had taken place in the past year.

### Knowledge, attitudes, perceptions and beliefs of healthcare workers

The vast majority of HCWs (93.4%) believed that RDTs provide certainty in diagnosing malaria, and 63.3% agreed or strongly agreed that RDTs can accurately diagnose the disease (Table 3). Similarly, 64.2% considered RDTs the best available method for malaria diagnosis, while 14.2% disagreed with this view. Confidence in performing RDTs was high; 89.8% agreed or strongly agreed that they could perform the test correctly, and 91.6% found RDTs easy to use. Most HCWs (85.8%) felt they had enough time to conduct the test, and 82.3% indicated they had sufficient time to wait for the results. In terms of resources, 84.5% reported having sufficient RDT supplies, and 65.9% stated that reference materials for consultation were available. However, nearly one in five HCWs (19.5%) disagreed with the availability of such reference materials, suggesting room for improvement in supporting documentation and guidance.

**Table 3:** Knowledge, attitudes and perceptions of healthcare workers

Characteristic	Response	Frequency	Percentage
RDT provides certainty of malaria	Yes	211	93.4
	No	15	6.6
RDTs can accurately diagnose malaria	Agree or strongly agree	143	63.3
	Neutral	47	20.8
	Disagree or strongly disagree	36	15.9
RDTs believed to be the best way to diagnose malaria	Agree or strongly agree	145	64.2
	Neutral	49	21.7
	Disagree or strongly disagree	32	14.2
Able to perform malaria RDT correctly	Agree or strongly agree	203	89.8
	Neutral	20	8.8
	Disagree or strongly disagree	3	1.3
RDTs considered easy to perform	Agree or strongly agree	207	91.6
	Neutral	18	8
	Disagree or strongly disagree	1	0.4
Have sufficient time to perform RDTs	Agree or strongly agree	194	85.8
	Neutral	23	10.2
	Disagree or strongly disagree	9	4
Have sufficient time to wait for RDT result	Agree or strongly agree	186	82.3
	Neutral	28	12.4
	Disagree or strongly disagree	12	5.3
Sufficient supplies for RDT	Agree or strongly agree	191	84.5
	Neutral	27	11.9
	Disagree or strongly disagree	8	3.5
Reference material for consultation available	Agree or strongly agree	149	65.9
	Neutral	33	14.6
	Disagree or strongly disagree	44	19.5
Is it possible for a patient to have a negative RDT test when they actually have a malaria infection?	Yes	202	89.4
	No	19	8.4
	I don’t know	5	2.2
Do you think you should treat a patient with an antimalarial even if their RDT returns a negative result?	Yes	44	19.5
	No	179	79.2
	I don’t know	3	1.3
Are you worried that a test is incorrect if the patient is febrile with a negative RDT?	Agree or strongly agree	70	31
	Neutral	47	20.8
	Disagree or strongly disagree	109	48.2
I use RDTs because the patients expect it	Agree or strongly agree	53	23.5
	Neutral	18	8
	Disagree or strongly disagree	155	68.6
I use RDTs because supervisors expect it	Agree or strongly agree	57	25.2
	Neutral	22	9.7
	Disagree or strongly disagree	147	65
I use RDTs because guidelines require it	Agree or strongly agree	136	60.2
	Neutral	29	12.8
	Disagree or strongly disagree	61	27
Other diagnostic tests for febrile illnesses available	Agree or strongly agree	107	47.3
	Neutral	25	11.1
	Disagree or strongly disagree	94	41.6
Medicines besides antimalarials available to treat febrile illness	Agree or strongly agree	167	73.9
	Neutral	28	12.4
	Disagree or strongly disagree	31	13.7

A large proportion of HCWs (89.4%) believed that a patient with a negative RDT could still have malaria (Table 3). However, only 19.5% believed that patients should still be treated with an antimalarial in such cases, while 79.2% disagreed with treating RDT-negative patients. Motivations for RDT use varied; 60.2% reported using RDTs because guidelines require it, while fewer HCWs cited external expectations as the main driver; only 25.2% agreed they used RDTs because supervisors expected it, and 23.5% said they used them because patients expected it. A majority (65–68.6%) disagreed that supervisor or patient expectations influenced their use of RDTs. Regarding the availability of alternative diagnostics and treatments, 47.3% agreed that other diagnostic tests for febrile illnesses were available at their facilities, while 41.6% disagreed. Most HCWs (73.9%) reported that medicines other than antimalarials were available for treating febrile illnesses.

### Healthcare workers’ proficiency in performing RDTs

Overall, HCWs demonstrated high proficiency in several key procedural, safety, and accuracy-related steps during the observed administration of RDTs (Table 4). All HCWs (100%) successfully opened the test package and collected an adequate amount of blood. Nearly all (99.5%) correctly pricked the finger with a sterile lancet and dispensed the blood in the appropriate well. A similar proportion (99.0%) properly cleaned the finger with alcohol and allowed it to dry beforehand.

**Table 4:** Proficiency of HCWs in performing an observed RDT

Proficiency of HCWs in performing an observed RDT	Frequency	Percentage
Assembles all materials (procedural)	150	73.2
Checks expiry date (accuracy)	83	40.5
Wears gloves (safety)	32	15.6
Opens the package and removes contents (procedural)	205	100.0
Writes the patients identifier on the RDT (procedural)	192	93.7
Explains the procedure to the patient (procedural)	90	43.9
Selects the correct finger for blood collection (procedural)	107	52.2
Cleans the finger with alcohol and allows to dry (safety)	203	99.0
Pricks finger firmly with sterile lancet (safety)	204	99.5
Discards lancet in sharps bin (safety)	155	75.6
Does not squeeze finger excessively (procedural)	125	61.0
Collects and adequate amount of blood (accuracy)	205	100.0
Dispenses blood in the appropriate well (accuracy)	204	99.5
Discards the pipette in the sharps bin (safety)	152	74.1
Dispenses the correct amount of buffer (accuracy)	185	90.2
Disposes of waste in appropriate container (procedural)	151	73.7
Waits for the appropriate time after adding buffer to read the result (accuracy)	83	40.5
Interprets the test correctly (accuracy)	200	97.6
Identifies the control line correctly (accuracy)	196	95.6

For accuracy, 97.6% of HCWs correctly interpreted the test results, and 95.6% accurately identified the control line. Most (90.2%) dispensed the correct amount of buffer, but only 40.5% waited the appropriate amount of time before reading the result, highlighting a key area for improvement. Checking the expiry date of the RDT, another critical accuracy step, was performed by just 40.5% of HCWs. In terms of procedural adherence, 93.7% wrote the patient identifier on the test, 73.7% disposed of waste appropriately, and 73.2% assembled all necessary materials before beginning. Only 61.0% avoided excessive squeezing of the finger, and just over half (52.2%) selected the correct finger for blood collection. Less than half (43.9%) explained the procedure to the patient. Safety practices showed mixed results. While nearly all HCWs (99.5%) pricked the finger with a sterile lancet and 75.6% discarded it in a sharps bin, only 15.6% wore gloves. Similarly, 74.1% correctly discarded the pipette in a sharps container.

These findings suggest strong overall performance in blood collection and test execution but point to weaknesses in timing, safety practices (especially glove use), and communication with patients that should be addressed through targeted training and supervision.

### Characteristics of RDTs observed in the study

Overall, the study team successfully captured 82% of all RDTs reported by the participating facilities. Of the 35,720 RDTs captured during the study period, 54.5% were recorded as positive and 45.5% as negative in the health facility registers (Table 5). The largest number of RDTs was performed in July (22.4%), aligning with the peak malaria transmission season. RDT testing remained relatively consistent from August through November, with each month accounting for approximately 15–19% of the total. Bioline-pf was the predominant RDT brand used, representing 99.4% of all tests. Very small proportions of First-response (0.01%) and Parahit (0.6%) RDTs were also used. Most patients tested were female (56.9%), and 40.3% were over the age of 15. Children under 5 years accounted for 33.9% of those tested, while 25.9% were aged 5–14 years. Malaria was diagnosed in 62.9% of patients tested. Over half (54.1%) of patients tested received ACTs, and 39.2% were prescribed antibiotics.

**Table 5:** Characteristics of RDTs observed in the study

Characteristic	Value	Frequency	Percentage
RDT result recorded in the health facility register	Negative	16,240	45.5
	Positive	19,480	54.5
Month RDT was performed	June*	3,108	8.7
	July	8,002	22.4
	August	5,578	15.6
	September	5,653	15.8
	October	6,865	19.2
	November	6,514	18.2
RDT brand	Bioline-pf	35,504	99.4
	First-response	3	0.01
	Parahit	213	0.6
Patient sex	Female	20,216	56.9
	Male	15,297	43.1
Patient age	< 5 years	12,099	33.9
	5_14 years	9,241	25.9
	> 15 years	14,379	40.3
Patient diagnosis	Malaria	22,486	63.0
Antimalarial medicine prescribed	ACT	19,341	54.1
Antibiotic medicine prescribed	Antibiotics	13,995	39.2

*Data from June represented only the latter half of the month (8.7%) due to the study start

### Agreement between results interpreted and recorded by healthcare workers and results interpreted by an external panel

#### Overall trends in agreement

**Fig. 3:**
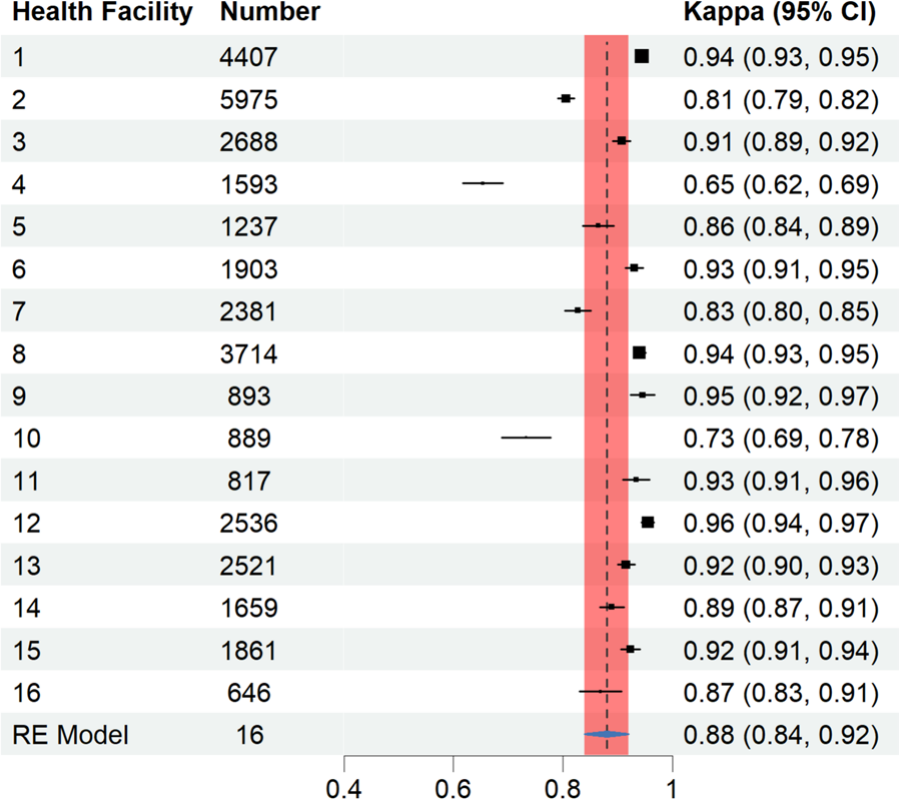
A forest plot of Cohen's kappa estimates by health facility and the overall estimate from the random-effects (RE) model

Overall, there was a high level of agreement (94.3%) between RDT interpretations recorded by HCWs and those of the external reference panel. Only 0.7% of positive results were misrecorded as negative, while 5.0% of negative results were misrecorded as positive (Table 6). Results misrecorded as positive were more common than those misrecorded as negative, a pattern that was consistent across different health facility and patient characteristics. The overall Cohen’s Kappa score was 0.88, indicating strong agreement between HCW and reference interpretations (Fig. 3). At the facility level, 13 out of 16 health facilities achieved a kappa score above 0.8, reflecting strong agreement, while three facilities had lower scores (< 0.8). In the Borgou Department, 6 of the 8 facilities achieved near-perfect agreement, as did 7 of the 8 facilities in the Zou Department.

**Table 6:** Overall agreement of HCWs RDT results with external panel result (n = 35,720)

Health care workers’ result	External panel result (Reference)		
	Positive	Negative	Invalid
Positive	Positive agreement 17,675 (49.5%)	Misrecorded as positive 1797 (5.0%)	Misrecorded as positive 8 (0.0%)
Negative	Misrecorded as negative 238 (0.7%)	Negative agreement 15,996 (44.8%)	Misrecorded as negative 6 (0.0%)

#### Agreement by health facility characteristics

Although overall agreement was high, the disaggregated analysis based on health facility characteristics revealed important variation by region, health zone, operational stratum, staffing, and infrastructure (Table 7).

**Table 7:** Agreement of HCWs and external panel RDT results by health facility characteristics

Characteristic	Characteristic	N	Agreement (%)	Kappa Score	Misrecorded as Positive (n, %)	Misrecorded as Negative (n, %)
Overall	Overall	35,720	94.3	0.88	1805 (5.1%)	244 (0.7%)
Region	Borgou	23,898	93.7	0.86	1383 (5.8%)	134 (0.6%)
	Zou	11,822	95.5	0.90	422 (3.6%)	110 (0.9%)
Health Zone	Bembèrèkè-Sinendé	16,477	94.1	0.88	883 (5.4%)	88 (0.5%)
	Bohicon-Za-Kpota-Zogbodomey	7564	96.4	0.90	223 (2.9%)	50 (0.7%)
	Djidja-Abomey-Agbangnizoun	4258	93.9	0.88	199 (4.7%)	60 (1.4%)
	Nikki-Kalalé-Pèrèrè	7421	92.6	0.84	500 (6.7%)	46 (0.6%)
Stratum	High volume/High positivity	10,906	91.1	0.83	894 (8.2%)	76 (0.7%)
	High volume/Low positivity	9812	96.8	0.93	236 (2.4%)	86 (0.9%)
	Low volume/High positivity	7179	94.9	0.86	311 (4.3%)	53 (0.7%)
	Low volume/Low positivity	7823	95.0	0.90	364 (4.7%)	29 (0.4%)
Parasite prevalence	20–29%	4407	97.2	0.91	104 (2.4%)	19 (0.4%)
	30–39%	31,313	93.8	0.87	1701 (5.4%)	225 (0.7%)
Number of staff performing RDTS	1–2 staff	817	96.7	0.93	22 (2.7%)	5 (0.6%)
	3–4 staff	5400	92.5	0.84	351 (6.7%)	43 (0.8%)
	5 + staff	29,503	94.5	0.89	1422 (4.8%)	196 (0.7%)
Internet access	Internet: No	6662	91.3	0.80	545 (8.2%)	37 (0.6%)
	Internet: Yes	29,058	95.0	0.90	1260 (4.3%)	207 (0.7%)
Main electricity source	Power: Grid	24,030	95.2	0.85	958 (4.0%)	194 (0.8%)
	Power: None	3027	92.6	0.84	215 (7.1%)	9 (0.3%)
	Power: Solar	8663	92.2	0.86	632 (7.3%)	41 (0.5%)

Regionally, HCWs in Zou achieved higher agreement (95.5%, Kappa: 0.90) than those in Borgou (93.7%, Kappa: 0.86). RDT results misrecorded as positives were lower in Zou (3.6%) than Borgou (5.8%), though Zou had slightly more results misrecorded as negatives (0.9% vs. 0.5%). Across health zones, Bohicon-Za-Kpota-Zogbodomey recorded the highest agreement (96.4%), while Nikki-Kalalé-Pèrèrè had the lowest (92.6%) and the highest rate of results miscrecorded as positive (6.7%). Djidja-Abomey-Agbangnizoun recorded the highest rate of results misrecorded as negative (1.4%).

Performance also varied across operational strata. Facilities with high volume but low TPRs showed the best results (96.8% agreement, Kappa: 0.93), while high volume/high positivity facilities had lower agreement (91.1%) and the highest rate of results misrecorded as positive (8.2%), suggesting that workload and disease burden can affect accuracy. By malaria prevalence, the single health facility with 20–29% prevalence showed the highest agreement (97.2%) and the lowest rates of misrecorded positives (2.4%) and misrecorded negatives (0.4%) while facilities with 30–39% prevalence had slightly lower agreement (93.9%) and higher misrecorded positives (5.4%).

Staffing levels also influenced performance. Facilities with one or two HCWs had the highest agreement (96.7%) and the lowest rate of results misrecorded as positive (2.7%). Facilities with three to four HCWs showed reduced agreement (92.6%) and higher misrecorded positives (6.7%), while those with larger teams had moderate agreement (94.6%). Infrastructure access had a clear impact. Facilities with internet access had higher agreement (95.0%) and fewer misrecorded positives (4.3%) than those without (91.3%, 8.1%). Facilities with grid electricity also performed better (95.2% agreement, 4.0% misrecorded positives) compared to those relying on solar (92.3%, 7.3%) or no electricity (92.6%, 7.1%).

These findings show that, while HCW performance in interpreting RDTs was generally high, diagnostic errors were more frequent in high-volume, high-burden, and infrastructure-limited settings.

#### Agreement by patient and HCW characteristics

Agreement between HCW-interpreted malaria RDT results and those of the reference panel, also varied across patient and HCW characteristics, occupational categories, and training levels (Table 8). Among patient characteristics, agreement was similar between females (94.1%) and males (94.6%), with slightly fewer misrecorded positives among males. RDT interpretation accuracy varied by patient age, with the highest agreement in children under five (96.7%) and the lowest in adults aged 15 and above (91.6%).

**Table 8:** Agreement of HCW and external panel RDT results by patient and HCWs’ characteristics

Characteristic	Variable	N	Positives	Negatives	Agreement (%)	Misrecorded Pos N (%)	Misrecorded Neg N (%)
Patient Sex	female	20,216	9507	10,703	19,019 (94.1%)	1057 (5.2%)	140 (0.7%)
	male	15,297	8299	6990	14,457 (94.6%)	737 (4.8%)	103 (0.7%)
Patient Age	< 5	12,099	6419	5673	11,691 (96.6%)	343 (2.8%)	65 (0.5%)
	5–14	9241	6815	2424	8814 (95.4%)	393 (4.3%)	34 (0.4%)
	15 +	14,379	4678	9696	13,165 (91.6%)	1069 (7.4%)	145 (1.0%)
HCW Sex	Female	19,654	9298	10,352	18,660 (94.9%)	854 (4.3%)	140 (0.7%)
	male	16,066	8615	7441	15,011 (93.4%)	951 (5.9%)	104 (0.6%)
HCW Age	< 30	12,953	6482		11,885 (91.8)	973 (7.5%)	95 (0.7%)
	30–39	9647	5258	4385	9062 (93.9%)	532 (5.5%)	53 (0.5%)
	40–49	9880	4574	5302	9563 (96.8%)	253 (2.6%)	64 (0.6%)
	50–59	3240	1599	1641	3161 (97.6%)	47 (1.5%)	32 (1%)
Occupational category	Community health worker	31	16	15	29 (93.5%)	2 (6.5%)	0 (0.0%)
	Medical auxiliary staff	15,843	7850	7987	14,973 (94.5%)	787 (5%)	83 (0.5%)
	Medical doctor	1738	1025	712	1641 (94.4%)	87 (5%)	10 (0.6%)
	Midwife	2062	823	1237	1890 (91.7%)	156 (7.6%)	16 (0.8%)
	Nurse	13,627	7052	6571	12,993 (95.3%)	540 (4%)	94 (0.7%)
	Student/intern/volunteer	2419	1147	1271	2145 (88.7%)	233 (9.6%)	41 (1.7%)
HCW qualification	Primary school or below	24,995	12,294	12,691	23,590 (94.4%)	1227 (4.9%)	178 (0.7%)
	Secondary school	7282	3718	3562	6829 (93.8%)	402 (5.5%)	51 (0.7%)
	University	3443	1901	1540	3252 (94.5%)	176 (5.1%)	15 (0.4%)
Years of experience	0–1 years	2218	1100	1117	2011 (90.7%)	189 (8.5%)	18 (0.8%)
	2–4 years	11,930	6098	5825	11,053 (92.6%)	799 (6.7%)	78 (0.7%)
	5–9 years	6238	3266	2972	5872 (94.1%)	329 (5.3%)	37 (0.6%)
	10 + years	15,334	7449	7879	14,735 (96.1%)	488 (3.2%)	111 (0.7%)
Average hours per week	0–19 h	1057	592	465	1006 (95.2%)	48 (4.5%)	3 (0.3%)
	20–39 h	452	233	219	429 (94.9%)	19 (4.2%)	4 (0.9%)
	40–49 h	5325	2906	2414	5092 (95.6%)	183 (3.4%)	50 (0.9%)
	> 50 h	28,886	14,182	14,695	27,144 (94.0%)	1555 (5.4%)	187 (0.6%)
Received onsite training in RDTs	No	15,726	7952	7766	14,751 (93.8%)	858 (5.5%)	117 (0.7%)
	Yes	19,994	9961	10,027	18,920 (94.6%)	947 (4.7%)	127 (0.6%)
Received onsite training in RDTs within past year	No	20,523	10,294	10,221	19,377 (94.4%)	996 (4.9%)	150 (0.7%)
	Yes, 2022 or 2023	15,197	7619	7572	14,294 (94.1%)	809 (5.3%)	94 (0.6%)

Among HCWs, females demonstrated slightly better performance than males (95.0% vs. 93.5% agreement), with fewer results misrecorded as positive. HCW performance also improved with age. Those aged 50–59 achieved the highest agreement (97.6%) and lowest misrecorded positives (1.5%). HCWs aged 30–39 had the lowest agreement (94.0%) and more misrecorded positives (5.5%). Among occupational groups, nurses and doctors showed high agreement (95.4% and 94.5%), while midwives and student/intern/volunteers performed less well, with student/interns having the highest misrecorded positive (9.6%) and misrecorded negative (1.7%) rates. Educational level made a small difference. HCWs with university education had slightly better performance than those with secondary school education. Agreement also increased with experience. HCWs with 10 or more years of experience had the highest agreement (96.1%) and lowest misrecorded positive rate (3.2%). Those with less than 5 years of experience showed more diagnostic errors.

Workload influenced accuracy; HCWs working over 50 h per week had slightly lower agreement (94%) and more misrecorded positives (5.4%) compared to those with lighter workloads. Training was beneficial; those who received onsite RDT training showed better agreement (94.7%) and fewer misrecorded positives (4.7%). However, recent onsite training (2022–2023) had almost no impact suggesting the importance of ongoing supervision and support.

#### Agreement by HCWs’ perceptions and beliefs

Agreement between HCW recorded RDT results and external panel readings varied according to HCWs’ knowledge, beliefs, and perceptions related to malaria diagnosis and RDT use (Table 9). HCWs who demonstrated correct knowledge such as knowing that the control line is essential for a valid test and that RDTs must be repeated if the control line is absent, had higher agreement rates (95.8% and 94.5%, respectively) and lower misrecorded positive results compared to those lacking this knowledge.

**Table 9:** Agreement of HCWs and external panel RDT results by patient and HCWs’ perceptions and beliefs

Characteristic	Variable	N	Positives	Negatives	Agreement (%)	Misrecorded Pos N (%)	Misrecorded Neg N (%)
Knows that the control line is required for an RDT test to be valid	no	25,726	12,907	12,819	24,107 (93.7%)	1438 (5.6)	181 (0.7%)
	Yes	9984	5006	4974	9564 (95.8%)	362 (3.6%)	58 (0.6%)
Knows an RDT test must be repeated if no control line is present	no	5669	2865	2804	5268 (92.9%)	361 (6.4%)	40 (0.7%)
	yes	30,048	15,048	14,989	28,403 (94.5%)	1443 (4.8%)	202 (0.7%)
Believes a patient with malaria can have a negative RDT	no	2794	1489	1305	2610 (93.4%)	165 (5.9%)	19 (0.7%)
	yes	32,249	16,063	16,173	30,425 (94.3%)	1601 (5%)	223 (0.7%)
Could provide antimalarial to a patient even if the RDT is negative	no	30,032	15,176	14,856	28,549 (95.1%)	1308 (4.4%)	175 (0.6%)
	yes	5567	2697	2866	5025 (90.3%)	477 (8.6%)	65 (1.2%)
Would be worried that a test is incorrect if patient is febrile but the RDT is negative	Disagree or strongly disagree	18,574	9624	8950	17,303 (93.2%)	1150 (6.2%)	121 (0.7%)
	Agree or strongly agree	5579	2646	2931	5215 (93.5%)	327 (5.9%)	37 (0.7%)
Uses RDTs because the patients expect it	Disagree or strongly disagree	25,175	12,903	12,272	23,759 (94.4%)	1241 (4.9%)	175 (0.7%)
	Agree or strongly agree	8588	4148	4437	8091 (94.2%)	447 (5.2%)	50 (0.6%)
Uses RDTs because the supervisors expect it	Disagree or strongly disagree	23,814	12,155	11,659	22,351 (93.9%)	1285 (5.4%)	178 (0.7%)
	Agree or strongly agree	8929	4260	4665	8468 (94.9%)	413 (4.6%)	48 (0.5%)
Uses RDTs because the guidelines require it	Disagree or strongly disagree	4932	2446	2486	4601 (93.3%)	267 (5.4%)	64 (1.3%)
	Agree or strongly agree	25,997	13,077	12,911	24,533 (94.8%)	1311 (5%)	153 (0.6%)
Other diagnostic tests for febrile illnesses are available	Disagree or strongly disagree	14,627	7301	7326	13,735 (93.9%)	780 (5.3%)	112 (0.8%)
	Agree or strongly agree	16,865	8570	8285	15,909 (94.4%)	842 (5%)	114 (0.7%)
Has access to medicines besides antimalarials available to treat febrile illness	Disagree or strongly disagree	4441	1943	2498	4275 (96.3%)	133 (3%)	33 (0.7%)
	Agree or strongly agree	25,996	13,254	12,730	24,415 (93.9%)	1418 (5.5%)	163 (0.6%)
RDT performance score	0–12	4438	1835	2601	4140 (93.3%)	253 (5.7%)	45 (1%)
	13–15	18,895	10,029	8858	17,674 (93.5%)	1096 (5.8%)	117 (0.6%)
	16–19	12,366	6035	6327	11,839 (95.7%)	447 (3.6%)	76 (0.6%)

Beliefs also influenced performance. HCWs who believed it is acceptable to give antimalarials despite a negative RDT had substantially lower agreement (90.3%) and higher rates of misrecorded positives (8.6%) and negatives (1.2%) than those who did not hold this belief. Higher agreement was observed among HCWs who reported using RDTs because supervisors expect it or clinical guidelines require it (94.9% and 94.8%, respectively), compared to those who disagreed with these statements (93.9% and 93.3%, respectively). Agreement was also higher among HCWs with access to alternative treatments for febrile illnesses (96.3%) and those with stronger RDT performance scores, which increased agreement from 93.3% among the lowest-scoring group to 95.7% among those scoring 16–19.

### Findings from health care worker in-depth interviews

*Characteristics of HCWs participating in in-depth interviews:* A total of 16 health care workers (HCWs) participated in the in-depth interviews, evenly split between high and low RDT agreement groups. Most were nursing assistants (11), had less than 15 years of experience (11), and were primarily involved in support roles. Fewer were directly engaged in malaria diagnosis (7) or treatment (6), with clinical responsibilities slightly more common among high agreement HCWs. Nearly all had received training in malaria services (14) and RDT use (13)..

*Use and Interpretation of RDTs:* Most HCWs demonstrated a clear understanding of the value of malaria RDTs and could accurately describe the procedures involved. While many found RDT interpretation straightforward, some reported challenges—particularly in reading faint results or distinguishing positive from negative outcomes. Both high and low agreement HCWs cited poor lighting, especially at night, as a barrier to accurate interpretation. Concerns were expressed when RDT results were negative despite clinical symptoms suggestive of malaria. In such cases, HCWs often repeated the test or referred the patient for microscopy, acknowledging that treatment without confirmation went against national guidelines. Some low agreement HCWs reported instances where microscopy confirmed malaria despite an initial negative RDT, leading to doubts about RDT reliability, particularly in cases of low parasitaemia.

“Sometimes we struggle to read the result after 15 min… We return later and see it’s positive.” — *High agreement nursing assistant, Tchikandou.*

“At night, we struggle to read the test correctly as the lights are too dim..….” — *High agreement nursing assistant, Tchikandou.*

“The RDT is effective, but sometimes it gives a negative result, and the thick blood smear is positive.” — *High agreement nursing assistant, Ouassaho.*

*Stockouts of RDTs and Supplies:* Although RDT and ACT stockouts were uncommon in the study sites, glove shortages were frequently reported. In the event of RDT stockouts, HCWs contacted nearby facilities to borrow supplies or resorted to microscopy or presumptive treatment. Responses to glove shortages varied; some HCWs asked patients to purchase gloves, while others proceeded without them, taking precautions to avoid direct contact with blood. This behavior did not differ significantly between HCW groups.

“When we run out of RDTs, we borrow or treat symptomatically while awaiting confirmation…” — *High agreement nursing assistant, Ouassaho.*

“Sometimes patients are asked to buy gloves. If not, we manage without touching the blood…” — *Low agreement nursing assistant, Bohicon.*

*Patient Volume and Workflow:* High patient loads, especially during the rainy season, were cited as a challenge. Nonetheless, most HCWs said they continued testing all suspected cases and prioritized urgent patients. No major differences in RDT interpretation were observed between high and low agreement HCWs during busy periods.

“We strive to perform all RDTs, regardless of patient numbers…” — *Low agreement nursing assistant, Bembèrèkè.*

“Severe cases are managed first, which can delay RDTs.” — *High agreement nursing assistant, Ouassaho.*

*Impact of Monthly Validation Exercises:* Most HCWs were aware of the monthly RDT validation exercises and participated in sorting used RDTs for review. The initiative was seen as supportive rather than burdensome. While high agreement HCWs reported that the exercise did not change their practices, lower agreement HCWs described it as helpful in aligning their work with national guidelines; suggesting it may be an important tool for quality improvement and data reliability.

“It motivates us to work more thoroughly.” — *Low agreement nursing assistant, Bohicon.*

“The validation hasn’t changed how I record results—I’ve always followed the guidelines.” — *High agreement nursing assistant, Agbokpa.*

## Discussion

This study provides one of the most comprehensive assessments to date of the accuracy of recording malaria RDT results in public health facilities in Benin. Across over 35,000 RDTs evaluated, there was a high level of agreement (94.3%) between interpretations recorded by HCWs and an external reference panel, with a Cohen’s Kappa score of 0.88 indicating strong reliability. These results highlight the overall accuracy of HCWs' interpretation and recording of RDT results under routine programmatic conditions and offer reassurance about the reliability of malaria data within the national HMIS. This should however be interpreted in the context of the ongoing nationwide decentralized monthly validation of malaria RDT results, introduced six months before the study, which may have contributed to improved HCW diagnostic practices by increasing scrutiny and accountability. The monthly validation involves a physical review and counting of positive and negative RDT cassettes recorded in health facility registers before the data are integrated into the HMIS [8, 14]. An interrupted time series analysis to investigate the impact of this strategy on malaria surveillance outcomes is underway.

Despite high overall agreement, we observed important variation in RDT interpretation accuracy. RDT results misrecorded as positives were significantly more common (5.0%) than those misrecorded as negative (0.7%), indicating a tendency among HCWs to deliberately override negative test results and treat presumptively. This finding is consistent with responses from the KAPB survey, where 89% of HCWs believed that a patient infected with malaria could still test negative on an RDT. This perception, commonly reported in other settings such as Kenya, Uganda, and Nigeria [10, 15–18], may erode HCWs’ confidence in negative results, leading to unnecessary antimalarial prescriptions and potential overuse of ACTs. HCWs’ non-adherence to negative RDT results has been extensively studied and is attributed to their awareness of key limitations of RDTs such as the risk of missing low-density infections and failure to detect non-falciparum species as well as the lack of alternative diagnostic tools or treatments, which may prompt precautionary antimalarial prescriptions for RDT-negative patients [10, 19, 20]. Training and supervision programmes designed to reinforce trust in negative results and build confidence in following test-based treatment protocols may be particularly impactful.

Interestingly, contrary to expectations that children under five, who are most vulnerable to malaria, might be more likely to be over-diagnosed, we found the highest accuracy and lowest error rates in this age group. Patients over 15 years of age had the lowest agreement and the highest rate of results misrecorded as positive (7.4%). This contradicts trends seen in other studies where caution may lead to overdiagnosis in young children [21]. One possible explanation is that adults are more likely to pressure HCWs into prescribing antimalarials despite negative results; a hypothesis also supported by qualitative interviews and findings from other contexts [9, 10, 22]. Variation in RDT recording accuracy was also observed across facility and HCW characteristics. Facilities with internet access and grid electricity had better performance than those lacking infrastructure, highlighting the role of basic facility readiness. High-volume, high-TPR settings were associated with lower agreement and higher error rates, suggesting that both workload and caseload intensity can compromise diagnostic accuracy. Among HCWs, performance improved with age and years of experience, with older HCWs and those with more than 10 years of experience achieving the highest agreement. Student interns and volunteers showed the poorest performance, reinforcing the importance of training and supervision for newer or less experienced cadres. The impact of HCW beliefs and motivations was also evident. HCWs who admitted they might treat despite a negative RDT result demonstrated the highest rate of results misrecorded as positive, suggesting that personal beliefs can undermine test-based treatment. Conversely, those who cited national guidelines or supervisory expectations as the reason for using RDTs tended to perform better. This suggests that accountability mechanisms and performance expectations may support improved practice.

While the study provides valuable insights, it was limited to peripheral public health facilities. These were selected because they serve the largest share of rural populations, where malaria burden is typically highest, and contribute the majority of routine HMIS data. They also benefit from stronger stock control and supervision systems compared to private providers. However, private health facilities and pharmacies also perform malaria diagnosis and contribute to national data. Given their more limited support from the Ministry of Health, diagnostic practices in private settings may be more variable. Future studies are recommended to assess RDT interpretation and reporting in private sector contexts to inform a broader national quality improvement strategy.

## Conclusion

This study found high accuracy in the interpretation and recording of malaria rapid diagnostic test (RDT) results by HCWs in public health facilities in Benin. Performance was strongest among HCWs with more experience, recent training, and access to supportive infrastructure such as internet and electricity. Nonetheless, efforts are needed to address persistent misrecorded positives, particularly among adult patients, and to reinforce HCWs’ confidence in reporting negative RDTs through targeted training and supportive supervision.

## Supplementary Information

Additional file1 (DOCX 23 KB)

## Data Availability

The datasets used and/or analyzed during the current study can be provided by the corresponding author on reasonable request.

## Abbreviations

ACT(s): Artemisinin-based combination therapy
AI: Artificial intelligence
CI: Confidence interval
CNERS: Comité National d’Éthique pour la Recherche en Santé
HCW: Health care worker
HCW(s): Health care worker(s)/Healthcare worker(s)
HMIS: Health management information system
KAPB: Knowledge, attitude, perception, and behavior/Knowledge, attitudes, perceptions, and behavior/Knowledge, attitudes, practices, and beliefs
MaCRA: Malaria RDT capturing and reporting assessment
NMCP: National Malaria Control Programme (Benin)
ODK: Open Data Kit
OPD: Outpatient department
Pe: Probability of expected (chance) agreement
PMC: Perennial malaria chemoprevention
Po: Probability of observed agreement
RDT: Rapid diagnostic test
SMC: Seasonal malaria chemoprevention
SSA: Sub-Saharan Africa
TPR: True positive rate
TPR(s): Test positivity rate(s)
WHO: World Health Organization
WCG IRB: Western Institutional Review Board
WMR: World Malaria Report
κ (kappa): Cohen’s kappa coefficient
