# Supplementary material for: The accuracy of recording malaria rapid diagnostic test (RDT) results in public health facilities in Benin; results from the MaCRA project
Source: Malar J. 2026 Mar 28;25:199. doi: 10.1186/s12936-026-05871-7 (PMC13151255; doi:10.1186/s12936-026-05871-7)
Supplement: Supplementary file 1 — Additional file1 (DOCX 23 KB) [file 12936_2026_5871_MOESM1_ESM.docx]

**The accuracy of recording malaria rapid diagnostic test (RDT) results in public health facilities in Benin; results from the MaCRA project.**

**Table S1. Infrastructure characteristics of 16 study facilities included in the MaCRA project, Benin**

| **Characteristic** | **Total** | **Percent** | **HF 1** | **HF 2** | **HF 3** | **HF 4** | **HF 5** | **HF 6** | **HF 7** | **HF 8** | **HF 9** | **HF 10** | **HF 11** | **HF 12** | **HF 13** | **HF 14** | **HF 15** | **HF 16** |  |
| --- | --- | --- | --- | --- | --- | --- | --- | --- | --- | --- | --- | --- | --- | --- | --- | --- | --- | --- | --- |
| **Internet access** | | | | | | | | | | | | | | | | | | | |
| Internet access | 13 | 81.25% | yes | yes | no | no | yes | yes | no | yes | yes | yes | yes | yes | yes | yes | yes | yes |  |
| Type of access |  |  |  |  |  |  |  |  |  |  |  |  |  |  |  |  |  |  |  |
| Mobile only | 5 | 31.25% | yes | no | no | no | no | no | no | no | no | no | no | no | yes | yes | yes | yes |  |
| Wifi only | 6 | 37.50% | no | no | no | no | yes | yes | no | no | yes | yes | yes | yes | no | no | no | no |  |
| Both mobile and wifi | 2 | 12.50% | no | yes | no | no | no | no | no | yes | no | no | no | no | no | no | no | no |  |
| **Reliability of internet access** | | | | | | | | | | | | | | | | | | | |
| Almost always available | 8 | 50% | yes | yes | no | no | no | yes | no | yes | yes | yes | no | no | yes | yes | no | no |  |
| Intermittent or rare | 5 | 31.25% | no | no | no | no | yes | no | no | no | no | no | yes | yes | no | no | yes | yes |  |
| No connection or coverage | 3 | 18.75% | no | no | yes | yes | no | no | yes | no | no | no | no | no | no | no | no | no |  |
| **Main power source** | | | | | | | | | | | | | | | | | | | |
| No power | 2 | 12.50% | no | no | no | no | no | no | yes | no | no | no | no | no | no | no | no | yes |  |
| Electrical grid | 12 | 75% | yes | no | no | yes | yes | yes | no | yes | yes | yes | yes | yes | yes | yes | yes | no |  |
| Solar | 2 | 12.50% | no | yes | yes | no | no | no | no | no | no | no | no | no | no | no | no | no |  |
| Generator | 0 | 0% | no | no | no | no | no | no | no | no | no | no | no | no | no | no | no | no |  |
| Other | 0 | 0% | no | no | no | no | no | no | no | no | no | no | no | no | no | no | no | no |  |
| **Water source** | | | | | | | | | | | | | | | | | | | |
| No water source | 6 | 37.50% | no | yes | yes | yes | yes | no | yes | no | no | no | no | no | no | yes | no | no |  |
| Rain water | 0 | 0% | no | no | no | no | no | no | no | no | no | no | no | no | no | no | no | no |  |
| Water source available | 10 | 62.50% | yes | no | no | no | no | yes | no | yes | yes | yes | yes | yes | yes | no | yes | yes |  |
| Borehole | 0 | 0% | no | no | no | no | no | no | no | no | no | no | no | no | no | no | no | no |  |
| Well | 0 | 0% | no | no | no | no | no | no | no | no | no | no | no | no | no | no | no | no |  |
| Creek, stream, river, spring | 2 | 12.50% | no | no | no | no | no | no | no | no | yes | yes | no | no | no | no | no | no |  |
| Pond | 0 | 0% | no | no | no | no | no | no | no | no | no | no | no | no | no | no | no | no |  |
| Bottled Water | 0 | 0% | no | no | no | no | no | no | no | no | no | no | no | no | no | no | no | no |  |
| Tap, piped | 8 | 50% | yes | no | no | no | no | yes | no | yes | no | no | yes | yes | yes | no | yes | yes |  |
| Other source | 0 | 0% | no | no | no | no | no | no | no | no | no | no | no | no | no | no | no | no |  |
